# Detection of Jingmenviruses in Japan with Evidence of Vertical Transmission in Ticks

**DOI:** 10.3390/v13122547

**Published:** 2021-12-19

**Authors:** Daisuke Kobayashi, Ryusei Kuwata, Toshiya Kimura, Hiroshi Shimoda, Ryosuke Fujita, Astri Nur Faizah, Izumi Kai, Ryo Matsumura, Yudai Kuroda, Shumpei Watanabe, Sawako Kuniyoshi, Takeo Yamauchi, Mamoru Watanabe, Yukiko Higa, Toshihiko Hayashi, Hiroto Shinomiya, Ken Maeda, Shinji Kasai, Kyoko Sawabe, Haruhiko Isawa

**Affiliations:** 1 Department of Medical Entomology, National Institute of Infectious Diseases, 1-23-1 Toyama, Shinjuku-ku, Tokyo 162-8640, Japan; dkoba@nih.go.jp (D.K.); r-fujita@agr.kyushu-u.ac.jp (R.F.); astrinf@nih.go.jp (A.N.F.); i-kai@niid.go.jp (I.K.); m-ryo@niid.go.jp (R.M.); tabanus-wata@titan.ocn.ne.jp (M.W.); saperoi@nih.go.jp (Y.H.); thaya@nih.go.jp (T.H.); kasacin@nih.go.jp (S.K.); sawabe@nih.go.jp (K.S.); 2 Faculty of Veterinary Medicine, Okayama University of Science, 1-3 Ikoinooka, Imabari City, Ehime 794-8555, Japan; r-kuwata@vet.ous.ac.jp (R.K.); s-watanabe@vet.ous.ac.jp (S.W.); 3 Yawatahama Public Health Center, 1-3-37 Kitahana, Yawatahama City, Ehime 796-0048, Japan; kimura-toshiya@pref.ehime.lg.jp; 4 Laboratory of Veterinary Microbiology, Joint Faculty of Veterinary Medicine, Yamaguchi University, 1677-1 Yoshida, Yamaguchi City, Yamaguchi 753-8515, Japan; hshimoda@yamaguchi-u.ac.jp; 5 Department of Veterinary Science, National Institute of Infectious Diseases, 1-23-1 Toyama, Shinjuku-ku, Tokyo 162-8640, Japan; ykuroda@niid.go.jp (Y.K.); kmaeda@nih.go.jp (K.M.); 6 Tsushima Wildlife Conservation Center, 2956-5 Nishizato, Sago, Kamigata-machi, Tsushima City, Nagasaki 817-1603, Japan; sawako.kuniyoshi1021@gmail.com; 7 Laboratory of Entomology, Obihiro University of Agriculture and Veterinary Medicine, Inada-cho, Obihiro City, Hokkaido 080-8555, Japan; tyamauchi@obihiro.ac.jp; 8 Ehime Prefectural Institute of Public Health and Environmental Science, 8-234 Sanban-cho, Matsuyama City, Ehime 790-0003, Japan; shinomiya-hiroto@pref.ehime.lg.jp

**Keywords:** Jingmen tick virus, Takachi virus, jingmenvirus, segmented flavi-like virus, tick-borne virus, arbovirus, virome, tick, Japan, emerging disease

## Abstract

Jingmen tick virus (JMTV) and the related jingmenvirus-termed Alongshan virus are recognized as globally emerging human pathogenic tick-borne viruses. These viruses have been detected in various mammals and invertebrates, although their natural transmission cycles remain unknown. JMTV and a novel jingmenvirus, tentatively named Takachi virus (TAKV), have now been identified during a surveillance of tick-borne viruses in Japan. JMTV was shown to be distributed across extensive areas of Japan and has been detected repeatedly at the same collection sites over several years, suggesting viral circulation in natural transmission cycles in these areas. Interestingly, these jingmenviruses may exist in a host tick species-specific manner. Vertical transmission of the virus in host ticks in nature was also indicated by the presence of JMTV in unfed host-questing *Amblyomma testudinarium* larvae. Further epidemiological surveillance and etiological studies are necessary to assess the status and risk of jingmenvirus infection in Japan.

## 1. Introduction

Ticks are hematophagous ectoparasites that infest various animals and can transmit a variety of pathogens, including protozoa, bacteria, and viruses, to their hosts during the blood-feeding process. Tick-borne viruses have become the focus of public health attention with the re-emergence of highly pathogenic viruses such as Crimean-Congo hemorrhagic fever virus (CCHFV) and the emergences of new viruses such as severe fever with thrombocytopenia syndrome virus and Bourbon virus [1].

Advances in next-generation sequencing (NGS) technologies have dramatically facilitated the study of tick-borne viruses. A series of recent studies have revealed the ticks harbor a wide variety of viruses through comprehensive analyses utilizing next-generation sequencers [2]. This has enabled the identification of novel viral sequences and virome analyses of ticks prior to the confirmation of infectivity and pathogenicity of these viruses in humans [3,4]. Therefore, NGS-based virome analysis of ticks has the potential to completely transform the elucidation of the etiology of unknown illness.

The detailed distribution of Jingmen tick virus (JMTV) is an example of successful virome analysis of ticks using next-generation sequencers. JMTV was first discovered in cattle ticks (*Rhipicephalus microplus*) collected in China in 2010 [5]. This virus has a four-segmented RNA genome, and, interestingly, several JMTV proteins are homologous to those of flaviviruses, a representative group of arthropod-borne viruses. The JMTV genome is predicted to encode five viral genes: nonstructural protein (NSP) 1, NSP2, viral protein (VP) 1, VP2, and VP3 that correspond to flaviviral NS5, NS3, envelope protein, core protein, and no known homolog, respectively [6]. Human infection with JMTV was first reported in fatal cases of Crimean-Congo hemorrhagic fever in Kosovo [7]. Later, the association between JMTV infection and clinical illness in humans was confirmed in China [8]. Currently, JMTV has been detected in cattle, monkeys, rodents, and bats and in humans (reviewed in Guo et al. [9]). Many studies have also used next-generation sequencers to detect JMTV in various tick species around the world (Table 1). To date, JMTV has been found to be distributed in Eurasia, Africa, and the Americas; this indicates a risk of JMTV infection across those broad areas.

Recently, a novel JMTV-related virus named Alongshan virus (ALSV) was identified from the blood of patients with a febrile illness in China [17]. These viruses, collectively called jingmenviruses, have been recently recognized as emerging tick-borne viruses [18]. Additionally, other viruses named Yanggou tick virus and Flavi-like segmented virus have been found in ticks and rodents, respectively, although their infectivity and pathogenicity to humans have not been clarified [19,20]. Jingmenviruses have attracted considerable interest as emerging tick-borne viruses. Although experimental animal infection models or reverse genetics systems for these viruses have not yet been established, their development is desirable to understand the basic properties of jingmenviruses [18]. Furthermore, JMTV and ALSV have also been detected in mosquitoes [5,17,21]; however, the mode of transmission of the viruses in nature is not well understood [18]. Therefore, elucidation of the natural history and transmission dynamics of these viruses is eagerly awaited for prevention and control of jingmenvirus infection.

Previously, we established and used an analytical method of examining RNA viromes in various hematophagous arthropods, including ticks [22,23,24,25,26,27,28]. Application of this method to routine tick-borne virus surveillance identified JMTV and its related virus sequences in field-caught ticks in various Japanese locations. Here, we report the results of the genetic and phylogenetic analyses of these viral sequences, and, for the first time, the possibility of vertical transmission of JMTV in their host ticks in nature.

## 2. Materials and Methods

### 2.1. Collection of Ticks

**Figure 1:**
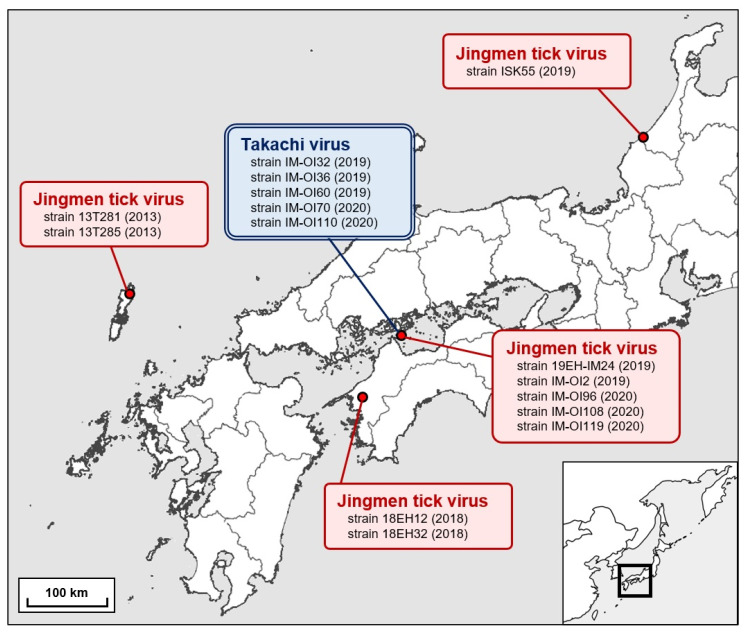
Collection sites of ticks positive for Jingmen tick virus (JMTV) and Takachi virus (TAKV) in Japan. The inset at the bottom right indicates the geographical location of the enlarged map. Red circles indicate sites where the virus-positive ticks were collected. The virus strain and sample collection year of JMTV (rounded rectangles with a red solid line) and TAKV (rounded rectangles with a blue double-lined border) are indicated.

Host-questing ticks were collected from vegetation fields in Ozu City and Ohshima Island of Imabari City, Ehime Prefecture, and in Wajima City and Kaga City, Ishikawa Prefecture, Japan, during 2018–2020 (Figure 1). Ticks were collected by dragging a flannel sheet (70 × 100 cm) across vegetation as described previously [23]. Collected ticks were classified into pools based on species, developmental stage, and sex and stored at −80 °C until further analyses.

### 2.2. RNA Virome Analyses Using Next-Generation Sequencer

Each pooled tick sample was homogenized with medium [Eagle’s Minimum Essential Medium (Sigma-Aldrich, St. Louis, MO, USA) with 2% heat-inactivated fetal bovine serum (Sigma-Aldrich), 200 U/mL penicillin (Thermo Fisher Scientific, Waltham, MA, USA), 200 μg/mL streptomycin (Thermo Fisher Scientific), and 5 μg/mL fungizone (Thermo Fisher Scientific)] and was passed through sterile 0.45-μm filters (Merck Millipore, Darmstadt, Germany), and then mixed in equal amounts up to a total of 380 µL (e.g., around 31.7 µL/pool for 12 samples). This sample was then used for library preparation for NGS analysis as described previously [25,26]. In brief, the filtrate was treated with three different types of nucleases [TURBO DNase (Thermo Fisher Scientific), Baseline-ZERO DNase (Lucigen, Middleton, WI, USA), and RNase A (Nippon Gene, Tokyo, Japan)], and RNA was extracted using ISOGEN II (Nippon Gene). cDNA libraries for NGS were prepared using NEBNext RNA first- and second-strand synthesis modules, NEBNext Ultra II End Repair/dA-Tailing Module, NEBNext Ultra II Ligation Module, and NEBNext Ultra II Q5 Master Mix (New England Biolabs, Ipswich, MA, USA). Prepared libraries were analyzed using the MiniSeq System with the MiniSeq Mid Output kit (300 cycles) (Illumina). Resulting reads were trimmed and *de novo* assembled using default settings on CLC Genomics Workbench version 21 (QIAGEN, Venlo, The Netherlands). Potential viral sequences were identified from the contigs through BLASTN and BLASTX searches against standard databases and non-redundant protein sequences database, respectively.

### 2.3. Virus Isolation

Attempts to isolate infectious virus from the aforementioned filtrated tick homogenates were made using Vero cells [African green monkey kidney, Japanese Collection of Research Bioresources Cell Bank (JCRB), Osaka, Japan] or BHK-21 cells (Syrian hamster kidney, JCRB) as described by Kobayashi et al. [23]. In brief, 50 µL of each filtrate of pooled tick homogenate prepared for RNA virome analysis described above was inoculated onto monolayer cells, which were incubated for 1 h at 37 °C and 5% CO_2_. A fresh culture medium was then added to each well, and the incubation was continued for seven days, followed by two subsequent blind passages under the same conditions. Culture supernatants after the incubation period were analyzed using next-generation sequencers (as described previously) [23,26] to confirm viral isolation.

### 2.4. Retrospective Screening of Samples from Previous Virus Isolation Studies

Previously, we used BHK-21 cells to isolate viruses from field-caught ticks from various parts of Japan during 2013 to 2014 [29]. The blind-passaged cell culture supernatants from 46 sample pools (Supplementary Table S1) were mixed (500 µL/pool), and the mixture concentrated and replaced with the SM buffer [50 mM Tris-HCl (pH 7.5), 100 mM NaCl, 8 mM MgSO_4_], as described previously [30]. The concentrated fluid was treated with nucleases and RNA was extracted as described previously [31]. Library preparation and analysis using next-generation sequencer were also conducted as described previously [31].

### 2.5. Determination and Characterization of Viral Genome Sequence

To identify virus-positive tick pools, reverse transcription polymerase chain reaction (RT-PCR) was performed on total RNA extracted from each filtered tick pool homogenate, as described previously [23]. RT-PCR was conducted using PrimeScript One Step RT-PCR Kit Ver. 2 (Takara Bio, Shiga, Japan) with the following two primer sets: JMTV-2F and JMTV-2R for JMTV, and IMOI-Js2-F and IMOI-Js2-R for a JMTV-like virus (Supplementary Table S2).

The virus-positive pools were then used to determine the complete viral genome sequence. Sequence gaps between contigs obtained using RNA virome analyses were filled with subsequent RT-PCRs using primers specific for the viral sequences. The resultant amplicons were analyzed using direct Sanger sequencing using an ABI 3130 Genetic Analyzer (Applied Biosystems, Waltham, MA) or Genewiz, Inc. (Saitama, Japan), as previously described [23,26]. The 3′ terminal sequences of each genome segment were amplified with the TaKaRa RNA PCR Kit (AMV) Ver. 3.0 (Takara Bio) using the provided Oligo dT-Adaptor Primer. Since the sequence and structure of the 5′ termini of each genome segment of known jingmenviruses remains to be determined, forward primers were designed based on the sequences closest to the 5′ termini of each genome segment obtained using RNA virome analysis (Supplementary Table S2). One-step RT-PCR was performed using the determined downstream sequences (Supplementary Table S2), and resulting amplicons were sequenced through the same procedure described above.

Jingmenviral open reading frames and their encoded amino acid sequences were determined using Genetyx version 14 software (Genetyx, Tokyo, Japan).

### 2.6. Examination of the Presence of Endogenous Viral Element (EVE)

Attempts to isolate Takachi virus (TAKV) in cell cultures failed, which raised the possibility that the TAKV sequence was derived from an EVE in the host tick genome. DNA extracted from the homogenate of TAKV-positive pool was analyzed through PCR using the following four virus-specific primer sets: IMOI-Js1-F and IMOI-Js1-R for segment 1, IMOI-Js2-F and IMOI-Js2-R for segment 2, IMOI-Js3-F and IMOI-Js3-R for segment 3, and IMOI-Js4-F and IMOI-Js4-R for segment 4 (Supplementary Table S2), as described previously [27,28]. The 18S ribosomal RNA gene was used as an internal positive control using the primer set T18S-F and T18S-R from our previous study [25].

### 2.7. Phylogenetic Analysis

Multiple sequence alignments were performed using the MAFFT online service [32]. The Gblocks program (version 0.91b, January 2002) [33] was used to remove divergent or ambiguously aligned regions, and the selection of the suitable substitution models and construction of the phylogenetic dendrograms were conducted using MEGA X [34].

## 3. Results

### 3.1. Detection of JMTV and Prevalence among Field-Collected Ticks in Japan

A total of 5008 ticks from at least nine tick species, including *Amblyomma testudinarium*, *Haemaphysalis flava*, *Hae. formosensis*, *Hae. hystricis*, *Hae. longicornis*, *Hae. megaspinosa*, *Haemaphysalis* sp., *Ixodes ovatus*, *I. persulcatus*, and *I. turdus*, were collected in 2018–2020 in Ehime and Ishikawa Prefectures, Japan (Supplementary Table S3). Tick samples were divided into 257 pools for RNA virome analyses. Using *de novo* assembly of the sequencing reads, several contigs of JMTV genome were identified through BLASTN analyses (data not shown). Eight pools derived from *Am. testudinarium* ticks were identified as JMTV-positive using RT-PCR screening (strain 18EH12, 18EH32, 19EH-IM24, IM-OI2, IM-OI96, IM-OI108, IM-OI119, ISK55; Table 2). After completion of sequence gaps between the viral contigs using virus-specific RT-PCRs, the JMTV genomic sequences, including the complete coding sequence, of a total of eight strains were determined. These sequence lengths were 3033 nucleotides (nt), 2747–2749 nt, 2738–2740 nt, and 2715–2717 nt, for segments 1, 2, 3, and 4, respectively. However, JMTV sequence was not detected in the blind-passaged supernatant of Vero or BHK-21 cells inoculated with these JMTV-positive pools (data not shown).

Conversely, a retrospective reanalysis of samples in our previous virus isolation studies (Supplementary Table S1) identified sequencing reads from JMTV. The lengths of the *de novo* assembled sequences were 742, 654, 624, and 822 nt for segments 1, 2, 3, and 4, respectively. Comparison of the nt sequence with those of other Japanese JMTV strains revealed a high sequence identity, at 95.87–99.19%, in all segments. Subsequent RT-PCR-based screening revealed that two independent pools derived from *Am. testudinarium* ticks were positive for JMTV. These ticks had been collected from the body surfaces of Amur leopard cats (*Prionailurus bengalensis euptilurus*), all of which were biting on the skin, in Tsushima City in 2013; the JMTV strains were named T281 and T285 (Table 2).

### 3.2. Phylogenetic Relationship between Japanese JMTV Strains and Other Known JMTV Strains

**Figure 2:**
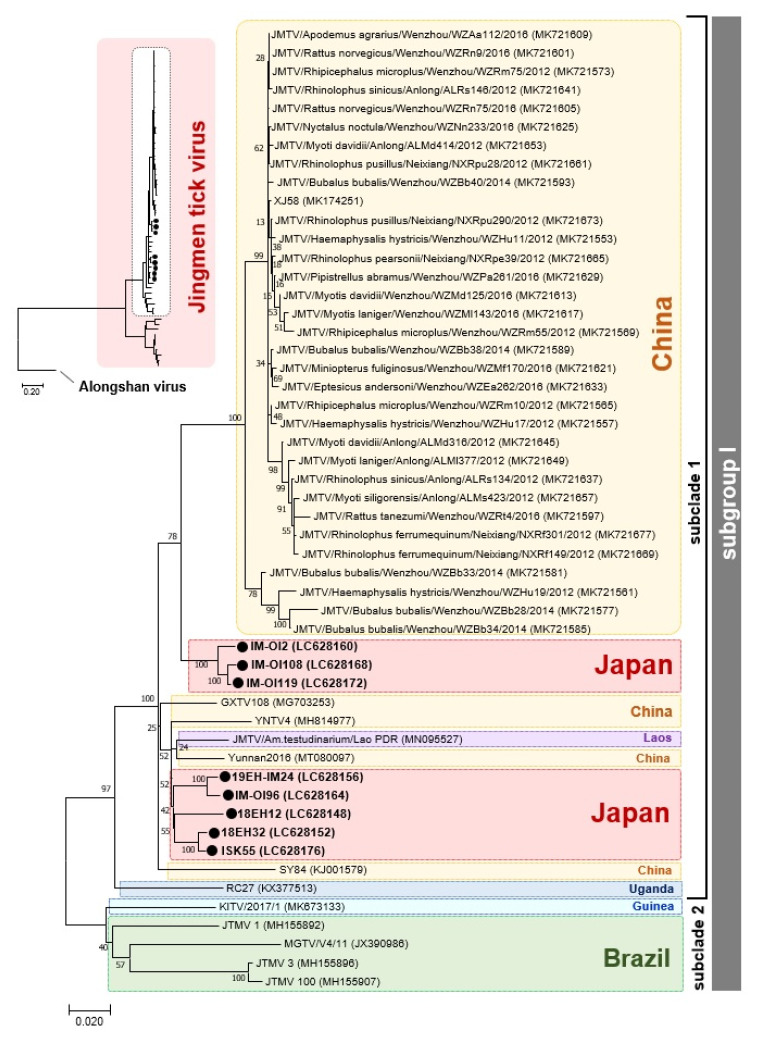
Phylogenetic relationships of Japanese strains of Jingmen tick virus (JMTV) with other known strains of JMTV. The dendrogram was constructed with nt sequences aligned through MAFFT FFT-NS-i using the maximum-likelihood method; the GTR + G + I model was employed for this analysis. The Alongshan virus strain Miass527 was used as the root for the tree. The phylogenetic tree shown on the left shows the dendrogram obtained from the analysis; the corresponding part of the JMTV subgroup I (surrounded by a dotted line) is enlarged on the right. In the dendrogram, the percentages of replicate trees where the associated taxa clustered together in the bootstrap test (1000 replicates) [35] are shown next to the branches. JMTV strains detected in this study are indicated in boldface and by filled circles. Accession numbers of the virus genome sequences used in this analysis are shown in parentheses. Countries where virus strains were detected are shown in the colored rounded rectangles with dotted lines.

We then used molecular phylogenetic analysis to investigate the phylogenetic relationships of the Japanese JMTV strains to other known strains. Phylogenetic dendrograms were constructed using alignments of the nt sequences of segment 1 of the Japanese strains with those of the known JMTV strains deposited in the DDBJ/EMBL/GenBank databases. All Japanese JMTV strains were grouped into subclade 1 in the JMTV subgroup I proposed by Guo et al. [9] (Figure 2). This subclade includes many Chinese strains and one strain each from Uganda and Laos [9]. Similarly, other segments of the Japanese JMTV strains were classified into the same subclade as the phylogeny of segment 1 (Supplementary Figure S1). However, in subclade 1, the phylogenetic positions of the Japanese JMTV strains differed with each strain and segment (Figure 1, Supplementary Figure S1). Overall, there was a close phylogenetic relationship between JMTV strains from Japan and those from the southern part of China (Yunnan Province and Guangxi Autonomous Region) and Laos.

Thus, this study uncovered a total of 10 JMTV strains from tick samples collected from a broad area in Japan (Figure 1). Interestingly, JMTV was detected only in *Am. testudinarium* ticks (Table 2). Notably, two strains of JMTV (IM-OI108 and IM-OI119) were detected in unfed host-questing larvae (Table 2), suggesting that the virus was transmitted vertically in the host ticks in nature.

### 3.3. Discovery of a Novel Jingmenvirus in Hae. Formosensis Ticks Collected in Japan

**Figure 3:**
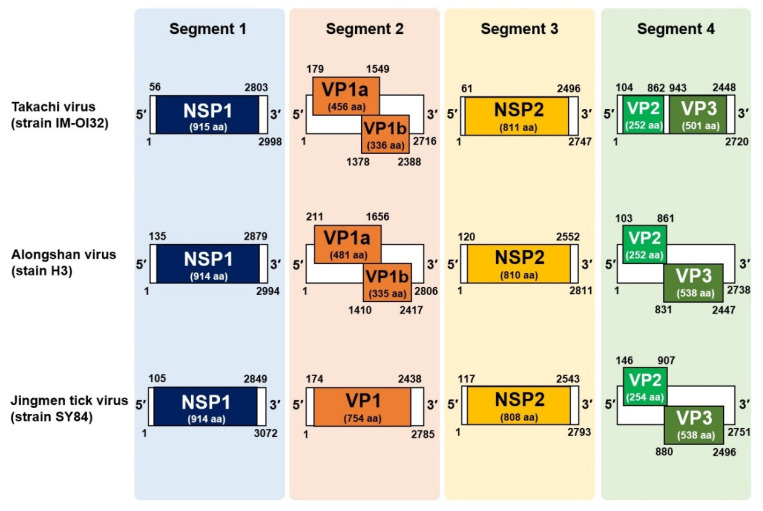
Comparison of genome structures of the jingmenviruses. Schematic illustrations of the genome organizations of three jingmenviruses, Alognshan virus, Jingmen tick virus, and Takachi virus. White and colored boxes represent viral genomes and open reading frames (ORF), respectively; numbers under the white and colored boxes denote the nt positions of the genome segments and each ORF, respectively. The name of each viral gene and the length of its amino-acid sequence are shown in the colored boxes.

During the virus surveillance, we detected additional JMTV-like sequences in five pooled samples of *Hae. formosensis* ticks (Table 2) that appeared to be derived from the same virus species. The lengths of sequences of all these strains were 2998, 2716, 2747, and 2720 nt for segments 1, 2, 3, and 4, respectively (Figure 3). The determined nt sequences shared 68–75% identity to ALSV through sequence comparison among the known jingmenviruses (data not shown). The predicted genome organization of the virus was essentially identical to those of JMTV and ALSV (Figure 3).

**Figure 4:**
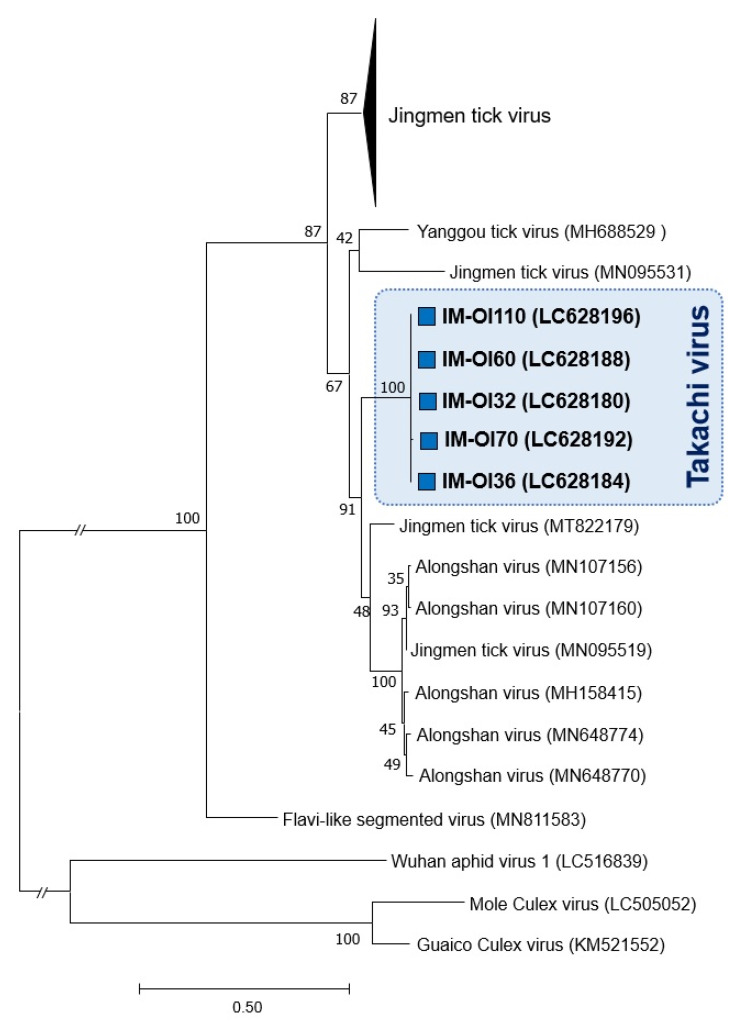
Phylogenetic relationships of a novel jingmenvirus, Takachi virus (TAKV), with other known strains of jingmenviruses. Amino-acid sequences of the nonstructural protein 1 (NSP1) were aligned using MAFFT L-INS-i. Divergent and ambiguously aligned regions were removed using Gblocks [33]. The dendrogram was constructed using the maximum-likelihood method with the LG + G model. Insect-associated jingmenviruses, including the Guaico Culex virus, Mole Culex virus, and Wuhan aphid virus 1, were used as the root of the tree. In both dendrograms, the percentages of replicate trees where the associated taxa clustered together in the bootstrap test (1000 replicates) [35] are shown next to the branches. TAKV is shown in boldface and by filled blue squares. Accession numbers of the virus genome sequences used in this analysis are shown in parentheses.

Phylogenetic analysis based on the amino acid sequences of NSP1 revealed that all viral strains were separated from the known viruses and formed a cluster with ALSV and Yanggou tick virus, a member of jingmenvirus detected in China and Russia [20] (Figure 4). Furthermore, using the same analyses with viral proteins (NSP2, VP1a, VP2, and VP3) showed the phylogenetic positions of the viruses to be similar to the result obtained from NSP1 (Supplementary Figure S2). These results suggest that this viral strain appears to be a novel jingmenvirus.

We then used DNA extraction and PCR to assess the possible presence of DNA forms of these viral sequences, such as an EVE. No PCR amplicons were detected using the viral gene-specific primer sets, although the amplicons of an internal control gene were observed (data not shown). Thus, the novel jingmenvirus genome likely exists as RNA but is not derived from the EVE in the host tick genome. Therefore, we tentatively named the new virus as Takachi virus (TAKV) after the Japanese name of the host tick species *Hae. formosensis*, “Takasago-chimadani.”

## 4. Discussion

Owing to the short history of the study of JMTV, there are still many unanswered questions about the natural transmission cycle of the virus. A previous study reported that JMTV has been detected in a variety of mammals and ticks at the same study site, the strains of which shared high sequence identities but were not clustered according to animal species in phylogenetic analysis [9]. Furthermore, JMTV strains with identical genomic sequences have sometimes been detected in both ticks and rodents, and therefore the authors claimed that these observations are compatible with the vector-borne transmission cycle of JMTV in nature [9]. Although JMTV has been detected in both ticks and mosquitoes [5,21], the JMTV transmission by mosquitoes has not yet been demonstrated. However, a study indicating JMTV accumulation in tick salivary glands and viral replication at the site of a tick bite in patient skin tissue strongly suggests that JMTV are tick borne [8]. Vector specificity of JMTV is poorly understood, and JMTV has been detected in a wide variety of tick species (17 tick species in 5 genera, Table 1). Most of the ticks in which JMTV has been detected were collected directly from animals (Table 1), and this raises the possibility that the detected JMTV is derived from the animal blood. Here, we only detected JMTV strains in *Am. testudinarium* ticks, although virus detection was performed on diverse unfed ticks from nine species across three genera. Remarkably, JMTV was detected not only in nymphs but also in unfed larva, indicating the vertical JMTV transmission in *Am. testudinarium* ticks in nature. In a previous study, an infectious JMTV was isolated from *Am. javanense*, and JMTV infection experiment in *Am. javanense* ticks has confirmed the virus accumulation and replication in the salivary glands, suggesting *Am. javanense* may be a potential vector of JMTV [8]; therefore, ticks from the *Amblyomma* genus may be important as vectors and reservoir hosts of JMTV. Three *Amblyomma* species are known to be distributed in Japan [36]. Among these, *Am. testudinarium*, where we detected JMTV in this study, is considered to be critical species as a JMTV vector because of its high preference for humans [37]. *Amblyomma testudinarium* is widely distributed from India to Southeast Asia and East Asia, including Japan (reviewed in Nakao et al. [38]); this suggests a possible distribution of JMTV in these areas.

The JMTV strains detected in this study were shown to be phylogenetically related to strains from southern China and Laos. This suggests that virus-infected ticks can be transported over a long distance by migratory birds as reported for CCHFV [39]. In Japan, two avian cases of *Am. testudinarium* infestation have been described in a resident bird species, *Scolopax mira* (reviewed in Yamauchi [40]), and in Korea, a migratory bird species, *Zoothera aurea*, has been reported to be parasitized by this tick species [41]. This tick-infested *Zoothera aurea* was captured in April, the migratory season from southern China or Southeast Asian region. The close phylogenetic relationship between JMTV strains from Japan and those from southern China and Laos implies that virus-infected ticks are transported by migratory birds over long distances. Alternatively, as *Am. testudinarium* ticks prefer to feed on wild boars [36], ticks may have migrated from the continent to the Japanese archipelago along with host animals, such as wild boars. Recently, the Japanese wild boar (*Sus scrofa leucomystax*) was shown to be genetically related to wild boar distributed in China and the Indochinese Peninsula, including Laos [42]. This study assumed that continental wild boars had migrated to and colonized the Japanese archipelago during cold periods when the sea level was low [42]. Furthermore, since JMTV has also been detected in cattle [5,14], it is possible that anthropogenic livestock movements can contribute to the migration and dispersal of the virus. These three possibilities involved in viral movement and dispersal (natural dispersal of migratory birds and land mammals and anthropogenic movement of livestock) have a very different time scale, and it is expected that further epidemiological and phylogenetic analysis of JMTV will reveal the natural transmission dynamics.

While attempting to isolate the virus, we detected JMTV RNA in the supernatant of BHK-21 cell cultures after two blind passages. Although this indicates possible virus replication in the cells, we cannot rule out the detection of viral RNA carryover from the virus-positive tick homogenates for inocula. According to the first report describing JMTV [5], JMTV genome was first detected in C6/36 and DH82 cells in the initial two passages after virus inoculation, but not after the following passages. On the other hand, another study demonstrated that ALSV, a close relative of JMTV, can be cultured for three years in tick-derived cultured cells (IRE/CTVM19 cell line) [20]. These differences in viral propagation in cultured cells may be related to differences in the doubling time of each cell line. The tick-derived IRE/CTVM19 cells used for the long-term culture of ALSV have a cell-doubling time of 10 days [43]. However, the doubling time of the cultured cells that allow a transient JMTV propagation, such as C6/36 and DH82 cells, has been reported to be approximately 0.9–2 days [44,45]. Indeed, the doubling time of BHK-21 or Vero cells used in this study was approximately 0.5–1 days [46,47]. These observations suggest that cells with a slow doubling time, such as tick-derived cultured cells, can provide a suitable environment for propagation of jingmenviruses, which have unusual multicomponent architectures, whereby genome segments that are separately enclosed in different particles must be incorporated into the same cell to enable complete replication of the virus [48]. Thus, the current study only detected two out of 10 JMTV strains from the BHK-21 or Vero cell culture supernatants after two blind passages. Therefore, further study is required to establish a more efficient method for isolation of JMTV and related viruses.

TAKV is a novel jingmenvirus that was discovered in this study and was only detected in *Hae. formosensis* ticks. This suggests a specific and stable virus-tick host relationship similar to those between JMTV and *Am. testudinarium* ticks, also demonstrated in this study. TAKV occupies a phylogenetically intermediate position between human-pathogenic JMTV and ALSV (Figure 4 and Supplementary Figure S2), raising the possibility that TAKV may be infectious and pathogenic to humans. *Hae. formosensis* is widely distributed in East and Southeast Asia and can parasitize a variety of mammals, including humans (reviewed in Kobayashi et al. [27]). Further investigations are required to determine the etiological aspect of this virus and evaluate the potential risk of human infection.

## 5. Conclusions

JMTV and a novel jingmenvirus, TAKV, were detected in host-questing ticks for the first time in Japan. JMTV was also detected in the supernatant of BHK-21 cells inoculated with the tick sample. Interestingly, these jingmenviruses may exist in a host-tick species-specific manner. This study also revealed that JMTV was distributed across an extensive area in Japan and was detected at the same collection site for several years. Moreover, JMTV was found in *Am. testudinarium* larvae, suggesting the vertical transmission of the virus in host ticks in nature. Further epidemiological surveillance and etiological studies are necessary to assess the status and risk of jingmenvirus infection in Japan.

## Figures and Tables

**Table 1:** List of tick species for which Jingmen tick virus was detected.

Genus	Species	Tick Hosts	Country	Reference
*Amblyomma*	*A. javanense*	Pangolin	China	[8]
	*A. testudinarium*	ND *	Laos	[10]
		NA (flagging) **	Japan	This study
*Haemaphysalis*	*Hae. campanulata*	Dog	China	[5]
	*Hae. flava*	Hedgehog, Badger	China	[5]
	*Hae. hystricis*	Mammals ***	China	[9]
		Badger	China	[5]
	*Hae. inermis*	Cattle	Turkey	[11]
	*Hae. longicornis*	Cattle, Dog, Goat	China	[5]
	*Hae. parva*	Cattle	Turkey	[11]
*Hyalomma*	*Hy. marginatum*	Cattle, Dog, Goat	Turkey	[11]
*Ixodes*	*I* *. granulatus*	ND	China	[5]
	*I. ricinus*	NA (flagging)	France	[10]
	*I. sinensis*	Wild goat	China	[5]
*Rhipicephalus*	*R. bursa*	Cattle, Goat, Sheep	Turkey	[11]
	*R. geigyi*	Cattle	Guinea	[12]
	*R. microplus*	Mammals	China	[9]
		Cattle	Brazil	[13]
		Cattle	Brazil	[14]
		Cattle	China	[5]
		Cattle or Buffalo	China	[15]
		Cattle	Trinidad and Tobago	[16]
		ND	French Antilles	[10]
	*R. sanguineus*	Dog	Turkey	[11]
		ND	China	[5]
	*R. turanicus*	Cattle	Turkey	[11]

* No direct description of collection methods or host animal species. ** Collected by dragging a flannel sheet, not from animals. *** Collected from dogs, goats, or cattle.

**Table 2:** Description of tick pools from which Jingmenviruses were detected in this study.

		Source			
Virus	Strain	Species	Stage and No. of Individuals	Collection Site	Collection Date
Jingmen tick virus	T281	*Amblyomma testudinarium*	9 larvae	Tsushima City, Nagasaki Prefecture, Japan	29 November 2013
	T285	*Amblyomma testudinarium*	6 nymphs	Tsushima City, Nagasaki Prefecture, Japan	15 December 2013
	18EH12	*Amblyomma testudinarium*	26 nymphs	Ozu City, Ehime Prefecture, Japan	27 September 2018
	18EH32	*Amblyomma testudinarium*	6 nymphs	Ozu City, Ehime Prefecture, Japan	26 September 2018
	19EH-IM24	*Amblyomma testudinarium*	7 nymphs	Imabari City, Ehime Prefecture, Japan	16 June 2019
	IM-OI2	*Amblyomma testudinarium*	5 nymphs	Imabari City, Ehime Prefecture, Japan	21 July 2019
	IM-OI96	*Amblyomma testudinarium*	5 nymphs	Imabari City, Ehime Prefecture, Japan	13 March 2020
	IM-OI108	*Amblyomma testudinarium*	4 larvae	Imabari City, Ehime Prefecture, Japan	6 May 2020
	IM-OI119	*Amblyomma testudinarium*	3 larvae	Imabari City, Ehime Prefecture, Japan	6 June 2020
	ISK55	*Amblyomma testudinarium*	1 nymph	Kaga City, Ishikawa Prefecture, Japan	23 April 2019
Takachi virus	IM-OI32	*Haemaphysalis formosensis*	42 nymphs	Imabari City, Ehime Prefecture, Japan	24 November 2019
	IM-OI36	*Haemaphysalis formosensis*	48 nymphs	Imabari City, Ehime Prefecture, Japan	3 December 2019
	IM-OI60	*Haemaphysalis formosensis*	50 nymphs	Imabari City, Ehime Prefecture, Japan	23 December 2019
	IM-OI70	*Haemaphysalis formosensis*	50 nymphs	Imabari City, Ehime Prefecture, Japan	17 January 2020
	IM-OI110	*Haemaphysalis formosensis*	50 nymphs	Imabari City, Ehime Prefecture, Japan	6 May 2020

## Data Availability

The genomic sequences of the JMTVs and TAKVs determined in this study have been deposited in the DDBJ/EMBL/GenBank databases under accession numbers LC628148-LC628199.

## Supplementary Materials

The following are available online at https://www.mdpi.com/article/10.3390/v13122547/s1, Figure S1: Phylogenetic relationship of Japanese and other strains of Jingmen tick virus. Figure S2: The phylogenetic relationship of Takachi virus (TAKV) and other jingmenviruses. Table S1: List of tick pools used for retrospective screening of isolated viruses obtained in our previous study. Table S2: List of primers used in this study. Table S3: Number of individuals of ticks used for RNA virome analyses in this study.
