# Supplementary material for: Detection of Jingmenviruses in Japan with Evidence of Vertical Transmission in Ticks
Source: Viruses. 2021 Dec 19;13(12):2547. doi: 10.3390/v13122547 (PMC8709010; doi:10.3390/v13122547)
Supplement: Supplementary file 1 [file viruses-13-02547-s001.zip › Supplementary Figures.pdf]

A

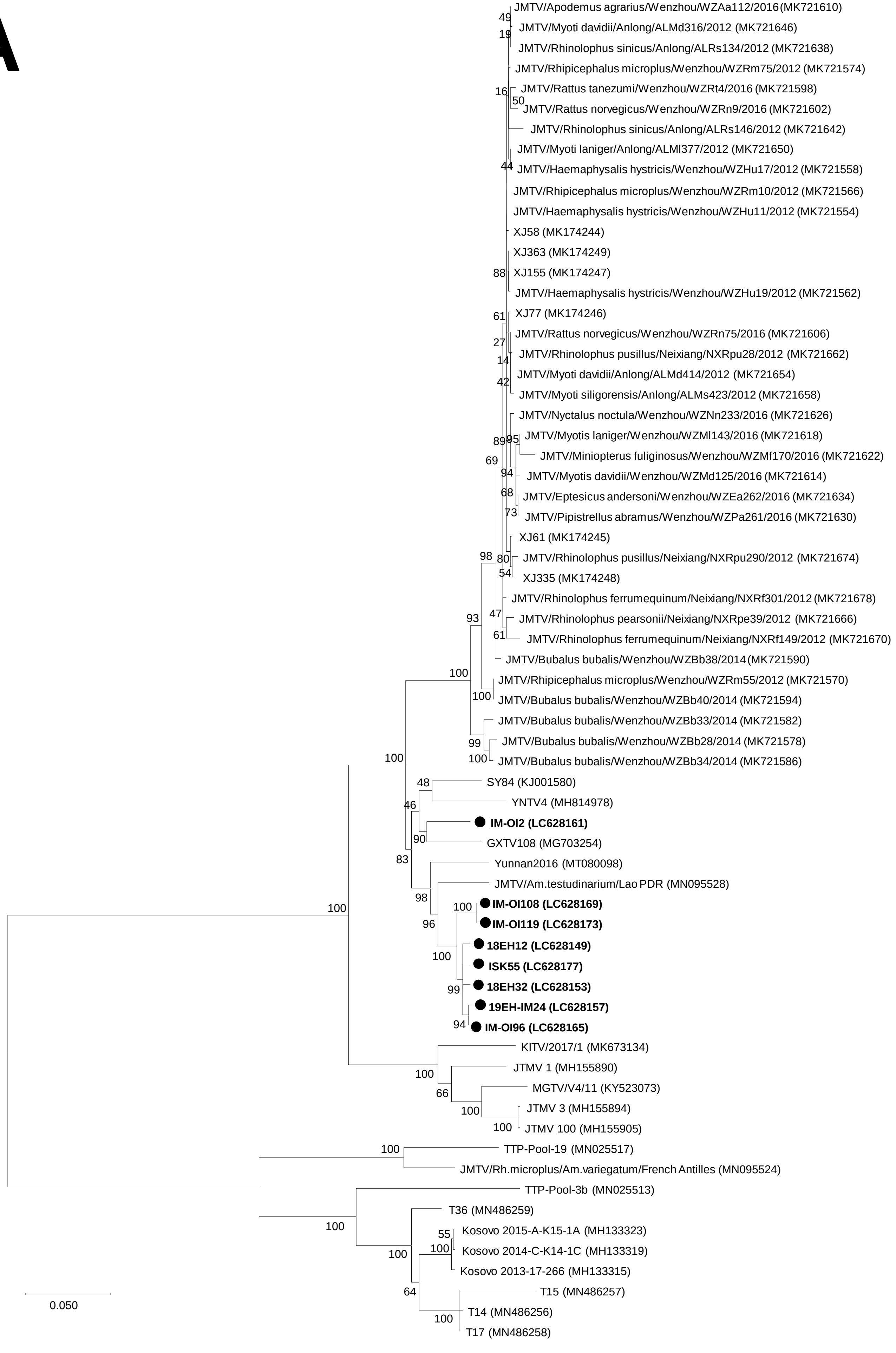

Supplementary Figure S1

B

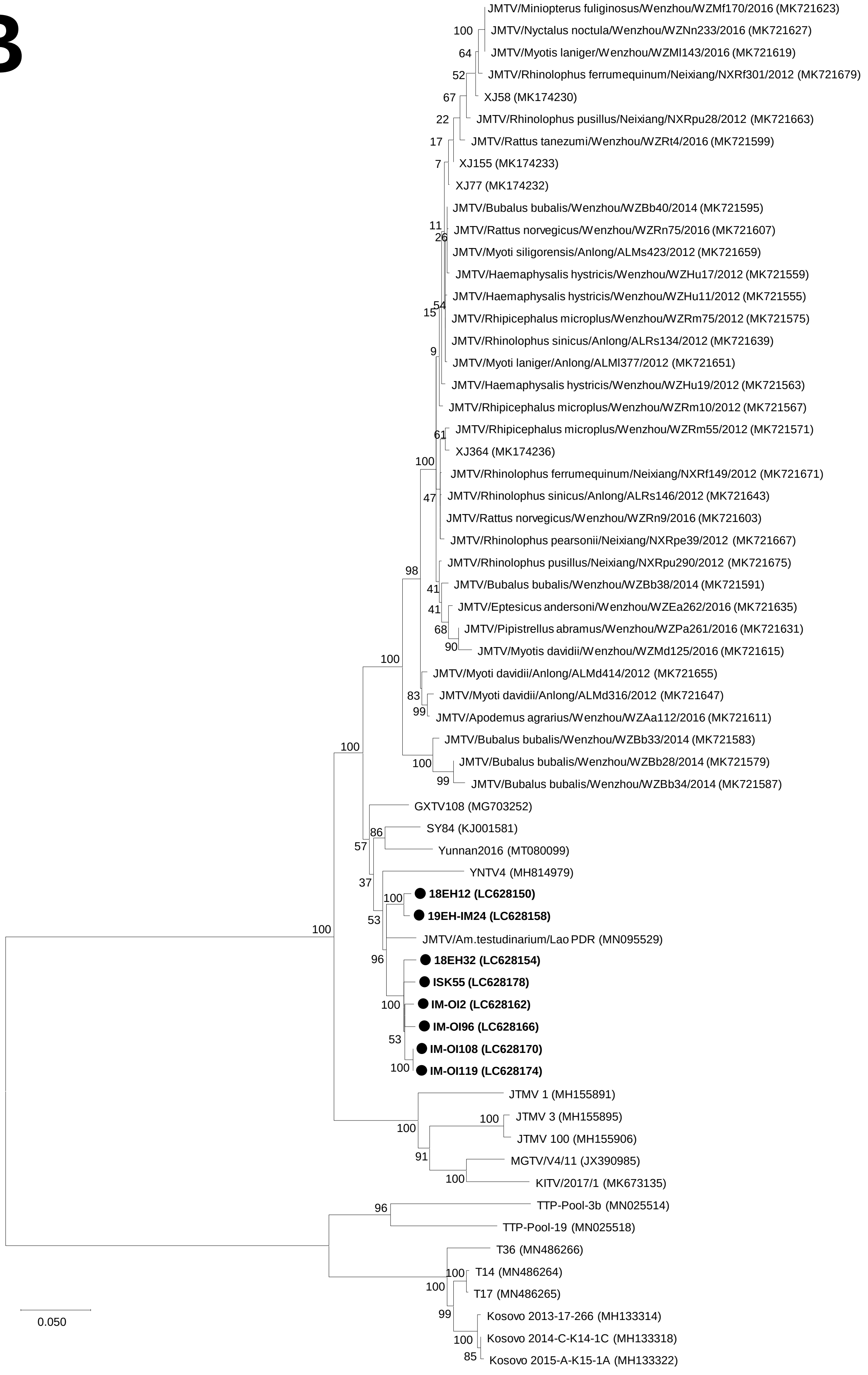

Supplementary Figure S1

C

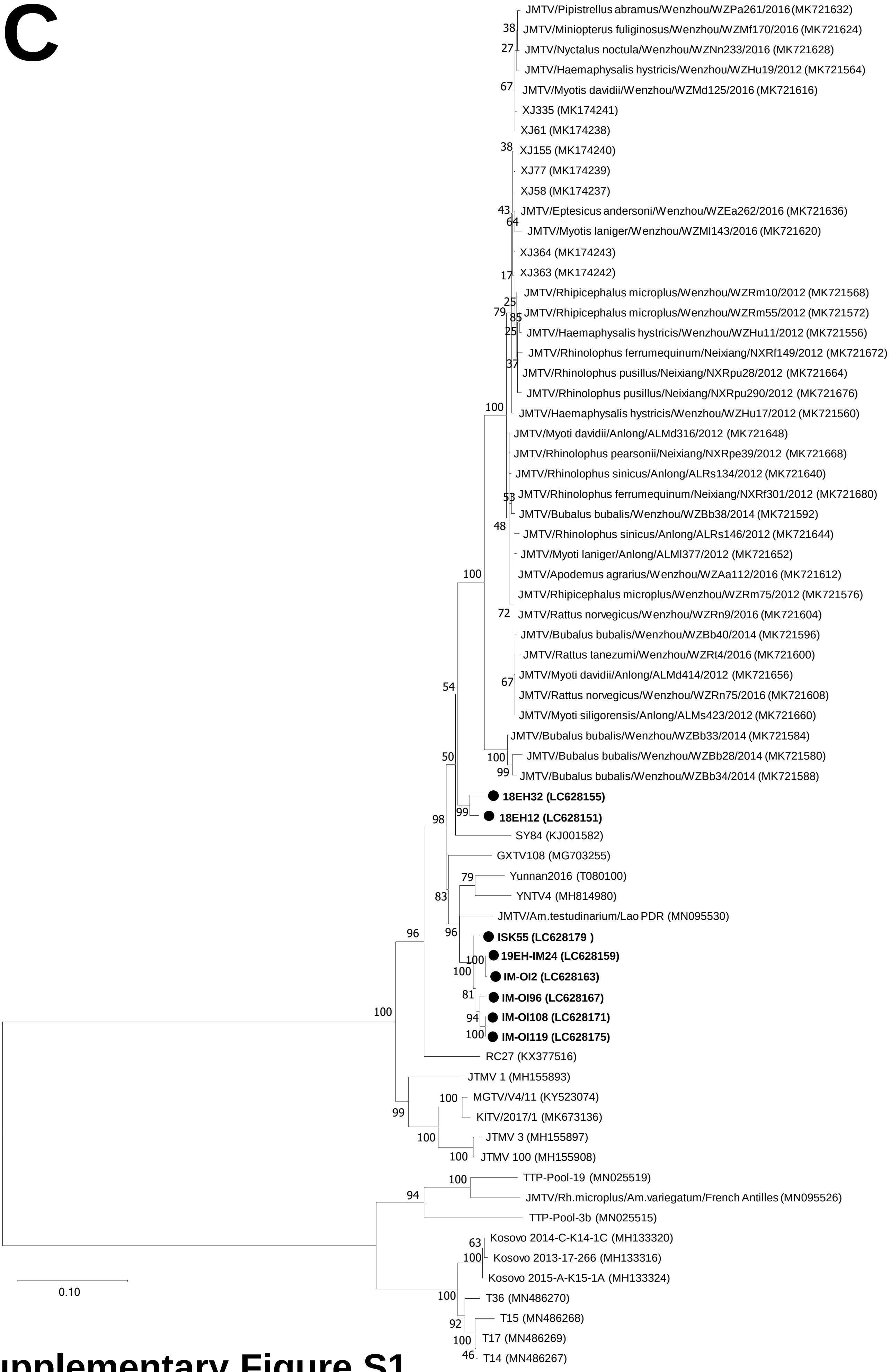

Supplementary Figure S1

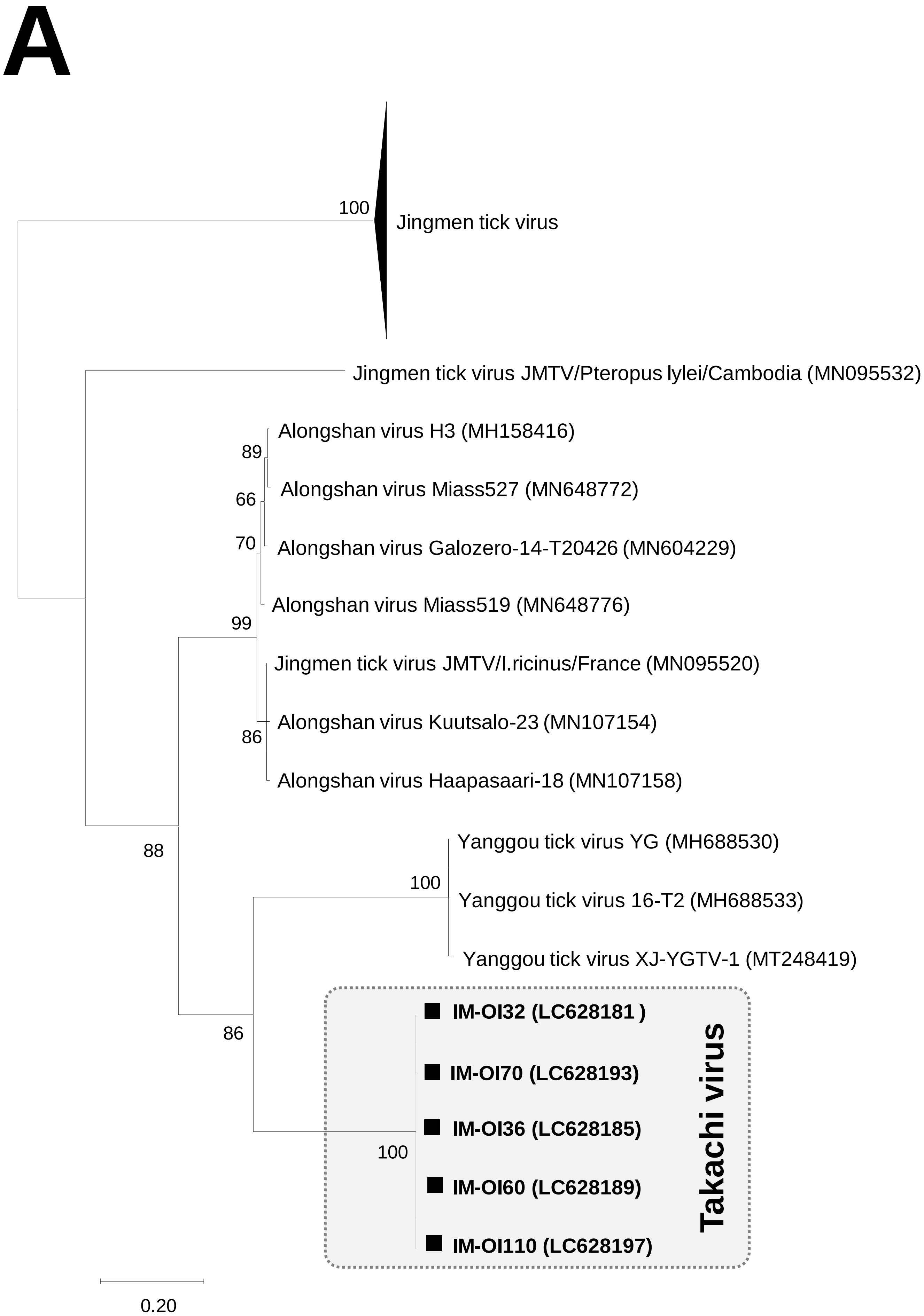

Supplementary Figure S2

B

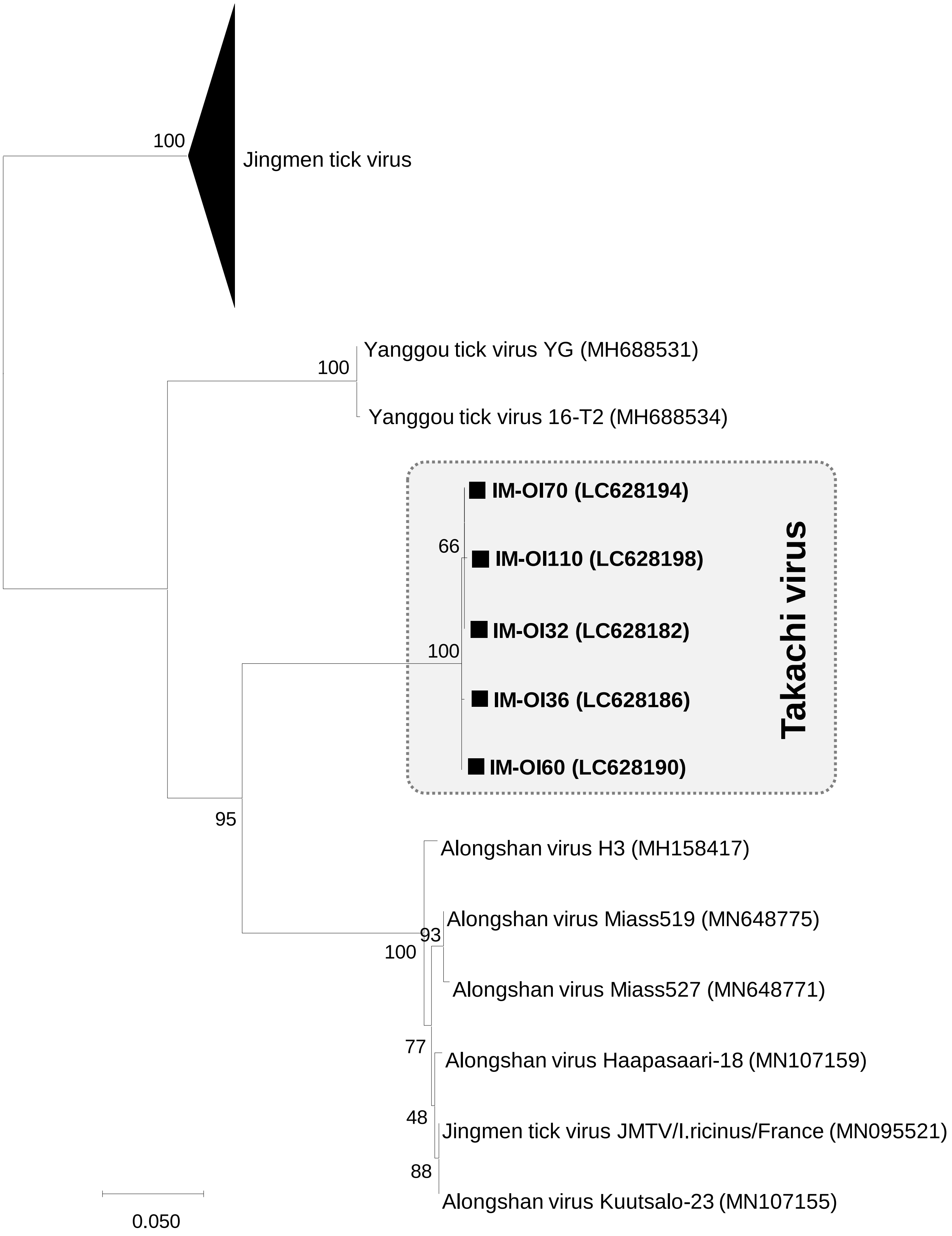

Supplementary Figure S2

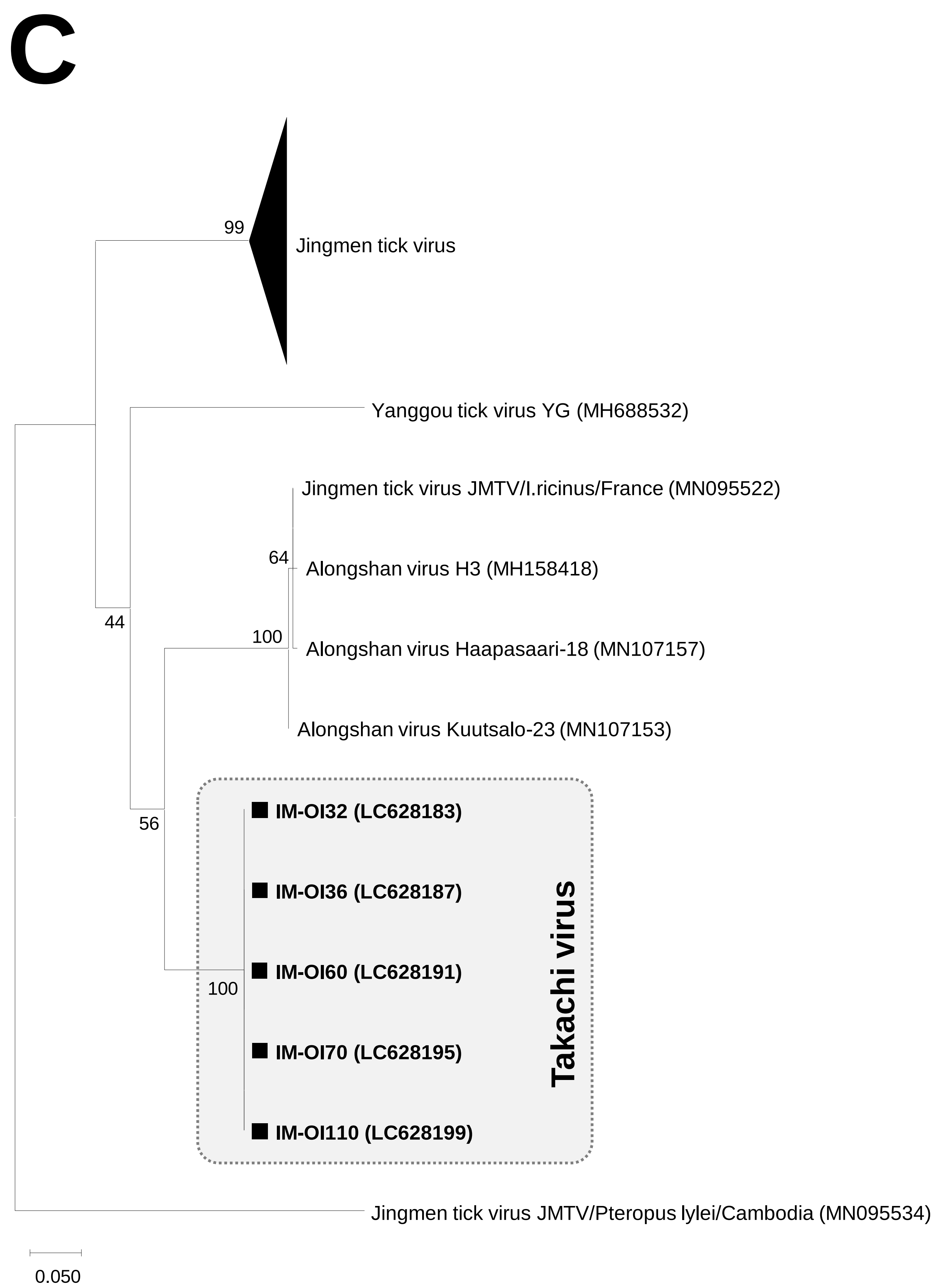

Supplementary Figure S2

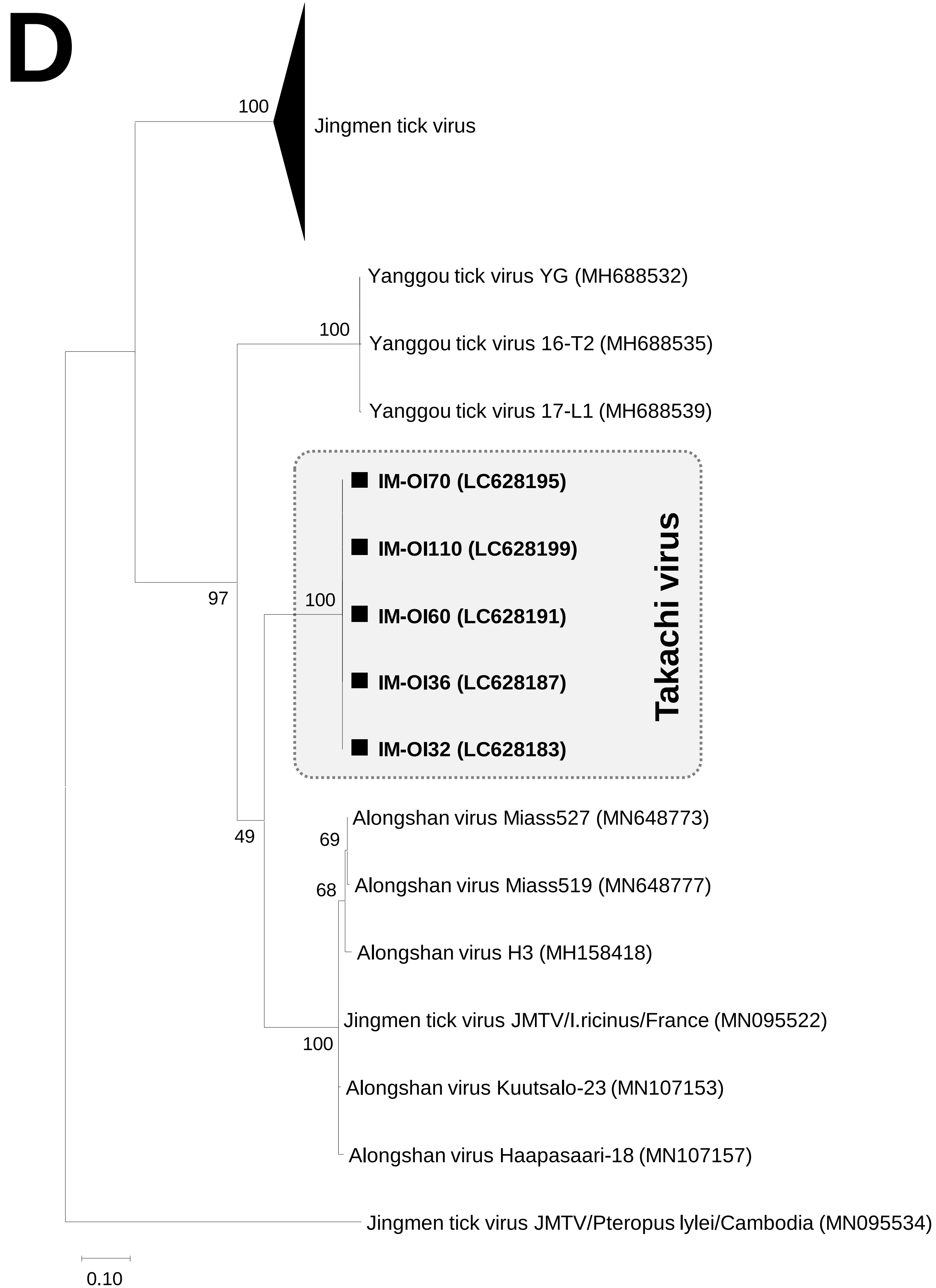

Supplementary Figure S2

Supplementary Figure Legend.

Supplementary Figure S1. Phylogenetic relationship of Japanese and other strains of Jingmen tick virus. The midpoint rooted phylogenetic dendrograms were constructed based on the nucleotide (nt) sequences of segments 2 (A), 3 (B), and 4 (C) using the maximum-likelihood method with the GTR + G + I model. The percentage of the replicate trees in which the associated taxa clustered together in the bootstrap test (1000 replicates) [1] is shown next to the branches. The selection of a suitable nt substitution model and construction of the phylogenetic dendrograms were performed on the MEGA X [2]. The strains detected in this study are indicated in boldface and preceded by a filled circle. The accession numbers of the virus genome sequences are shown in brackets.

Supplementary Figure S2. The phylogenetic relationship of Takachi virus (TAKV) and other jingmenviruses. The midpoint rooted phylogenetic dendrograms were constructed based on the conserved amino acid sequence of the VP1a (A), NSP2 (B), VP2 (C), and VP3 (D) using the maximum-likelihood method. The amino acid substitution models used in each dendrogram construction were JTT + G + F (for NSP2), JTT + G (VP1a and VP3), and LG + G (VP2). The percentage of replicate trees in which the associated taxa clustered together in the bootstrap test (1000 replicates) [1] is shown next to the branches. The selection of a suitable amino acid sequence substitution model and construction of the phylogenetic dendrograms were performed on the MEGA X [2]. TAKV is indicated by gray rounded rectangles with a dotted line, and the strains are indicated as in boldface preceded by a filled square. The accession numbers of virus genome sequences are shown in brackets.

References

1. Felsenstein, J. Confidence limits on phylogenies: an approach using the bootstrap. *Evolution.* **1985**, *39*, 783–791. doi: 10.1111/j.1558-5646.1985.tb00420.x.
2. Kumar, S.; Stecher, G.; Li, M.; Knyaz, C.; Tamura, K. MEGA X: Molecular evolutionary genetics analysis across computing platforms. *Mol. Biol. Evol.* **2018**, *35*, 1547–1549. doi: 10.1093/molbev/msy096.
