# Supplementary material for: Detection of Jingmenviruses in Japan with Evidence of Vertical Transmission in Ticks
Source: Viruses. 2021 Dec 19;13(12):2547. doi: 10.3390/v13122547 (PMC8709010; doi:10.3390/v13122547)
Supplement: Supplementary file 1 [file viruses-13-02547-s001.zip › Supplementary Tables_v2.pdf]

Supplementary Table S1. List of tick pools used for retrospective screening of isolated viruses obtained in our previous study.

			No. of individuals					
No.	Pool no.	Species	Adult female	Adult male	Nymph	Larva	Collection date	Collection site
1	T241	*Haemaphysalis flava*	2	1	15		20 January, 2014	Fukiage-cho, Hioki City, Kagoshima Prefecture, Japan
2	T242	*Haemaphysalis formosensis*			3		20 January, 2014	Fukiage-cho, Hioki City, Kagoshima Prefecture, Japan
3	T243	*Haemaphysalis flava*		1	14		26 January, 2014	Odawara City, Kanagawa Prefecture, Japan
4	T244	*Ixodes turdus*	1			8	26 January, 2014	Odawara City, Kanagawa Prefecture, Japan
5	T245	*Ixodes ovatus*		1			21 July, 2013	Minokoshi, Mt. Tsurugi, Tokushima Prefecture, Japan
6	T246	*Haemaphysalis flava*			1		3 September, 2013	Yoshino-cho, Ishinomaki City, Miyagi Prefecture, Japan
7	T247	*Haemaphysalis longicornis*	1		1		9 July and 3 September, 2013	Yoshino-cho, Ishinomaki City, Miyagi Prefecture, Japan
8	T248	*Haemaphysalis longicornis*	3		2		10 July, 7 August, and 6 September, 2013	Takata-cho, Rikuzentakata City, Iwate Prefecture, Japan
9	T249	*Haemaphysalis megaspinosa*			4		10 July, 2013	Takata-cho, Rikuzentakata City, Iwate Prefecture, Japan
10	T250	*Haemaphysalis flava*		1			6 September, 2013	Takata-cho, Rikuzentakata City, Iwate Prefecture, Japan
11	T251	*Haemaphysalis megaspinosa*			3		7 August, 2013	Takata-cho, Rikuzentakata City, Iwate Prefecture, Japan
12	T252	*Haemaphysalis flava*	1		10		30 October, 2013	Mt. Matsukira, Mino City, Gifu Prefecture, Japan
13	T253	*Haemaphysalis kitaokai*	2				17 November, 2013	Shimobayashi-machi, Takayama City, Gifu Prefecture, Japan
14	T254	*Haemaphysalis flava*	2	3	29		7 June, 2013	Hachioji City, Tokyo, Japan
15	T255	*Haemaphysalis longicornis*			1		15 June, 2013	Kanmami-cho, Shizuoka Prefecture, Japan
16	T256	*Haemaphysalis longicornis*	1		14		24 May, 2013	Hachioji City, Tokyo, Japan
17	T257	*Haemaphysalis flava*	1	2	36		24 May, 2013	Hachioji City, Tokyo, Japan
18	T258	*Haemaphysalis megaspinosa*	1		4		24 May, 2013	Hachioji City, Tokyo, Japan
19	T259	*Haemaphysalis longicornis*	1		5		31 May, 2013	Hachioji City, Tokyo, Japan
20	T260	*Haemaphysalis flava*	2	2	26		26 May, 2013	Hachioji City, Tokyo, Japan
21	T261	*Haemaphysalis longicornis*	2		18		7 June, 2013	Hachioji City, Tokyo, Japan
22	T262	*Haemaphysalis longicornis*	6		21		24 and 28 June, 2013	Hachioji City, Tokyo, Japan
23	T263	*Haemaphysalis flava*		1	20		24 and 28 June, 2013	Hachioji City, Tokyo, Japan
24	T264	*Haemaphysalis megaspinosa*	1	1			20 October, 2013	Hiroshima City, Hiroshima Prefecture, Japan
25	T265	*Haemaphysalis flava*	1		4		16 November, 2013	Hiroshima City, Hiroshima Prefecture, Japan
26	T266	*Haemaphysalis kitaokai*	3				2 and 16 November, 2013	Hiroshima City, Hiroshima Prefecture, Japan

27	T267	*Haemaphysalis megaspinosa*		3	6		2 and 16 November, 2013	Hiroshima City, Hiroshima Prefecture, Japan
28	T268	*Ixodes turdus*			1		25 January, 2014	Hiroshima City, Hiroshima Prefecture, Japan
29	T269	*Haemaphysalis flava*	2	3	22		12 and 19 October, 2013	Yonago City and Yodoe-cho, Tottori Prefecture, Japan
30	T270	*Amblyomma testudinarium*			1		19 October, 2013	Kiyomizu-cho, Yasugi City, Shimane Prefecture, Japan
31	T271	*Haemaphysalis flava*			29		19 October, 2013	Kiyomizu-cho, Yasugi City, Shimane Prefecture, Japan
32	T272	*Haemaphysalis megaspinosa*			1		19 October, 2013	Kiyomizu-cho, Yasugi City, Shimane Prefecture, Japan
33	T273	*Haemaphysalis flava*			20		10 Septembar, 2013	Tsubata-machi, Ishikawa Prefecture, Japan
34	T274	*Haemaphysalis flava*	5	9	11		17 October, 2013	Tsubata-machi, Ishikawa Prefecture, Japan
35	T275	*Haemaphysalis flava*	5		22		24 Septembar and 2 Novembar, 2013	Wajima City, Ishikawa Prefecture, Japan
36	T276	*Haemaphysalis flava*	1	1	22		8 November, 2013	Soma City, Fukushima Prefecture, Japan
37	T277	*Haemaphysalis flava*	5	1	6		29 October, 2013	Nagaoka City, Niigata Prefecture, Japan
38	T278	*Haemaphysalis flava*	5	6			29 October, 2013	Kashiwazaki City, Niigata Prefecture, Japan
39	T279	*Haemaphysalis flava*	1	2	6		21 October, 2013	Nagano City, Nagano Prefecture, Japan
40	T280	*Haemaphysalis flava*	3	4			20 November, 2013	Tsushima City, Nagasaki Prefecture, Japan
41	T281	*Amblyomma testudinarium*				9	29 November, 2013	Tsushima City, Nagasaki Prefecture, Japan
42	T282	*Amblyomma testudinarium*			1		5 November, 2013	Tsushima City, Nagasaki Prefecture, Japan
43	T283	*Ixodes tanuki*		3			1 December, 2013	Tsushima City, Nagasaki Prefecture, Japan
44	T284	*Amblyomma testudinarium*			1		5 December, 2013	Tsushima City, Nagasaki Prefecture, Japan
45	T285	*Amblyomma testudinarium*			6		15 December, 2013	Tsushima City, Nagasaki Prefecture, Japan
46	T286	*Haemaphysalis megaspinosa*	1	1			30 December, 2013	Tsushima City, Nagasaki Prefecture, Japan

Supplementary Table S2. List of primers used in this study.

Virus	Segment	Primer name	Sequence (5'-3')
Jingmen tick virus	1	JMTV-1F	GAGAGAGGCAGAGAGGAATGGAT
	1	JMTV-1R	TTCTTGGTCTTCACTTGCCACTT
	1	JMTV-s1-3GSP1	GGCTCTKGCCATCCTCTCGGTAA
	1	JMTV-s1-5GSP1	ACCACTCCCCTGAACTTCATTCT
	2	JMTV-2F	GGCGGTTCATCCTTTAACACTGT
	2	JMTV-2R	AGGGACCACCCAGAACCTCCACT
	2	JMTV-s2-3GSP1	GACATTCGAATGCGGAACGACAT
	2	JMTV-s2-5GSP1	GCGAGAGCTGCGCGCGAAAGAAT
	3	JMTV-3F	GGCCCTGAGATGATACAAGGAGC
	3	JMTV-3R	GCTAGGGCGCCAGTGTTTGTCTT
	3	JMTV-s3-3GSP1	GTGGCTGAGCAGCGGGAGGAATA
	3	JMTV-s3-5GSP1	ATAGTGGGCAGCAGCTCCATGAA
	4	JMTV-4F	TTCGCAGGCACGTTTGTGATGGT
	4	JMTV-4R	GTGCGGTTACGCTACCTCCGCAA
	4	JMTV-s4-3GSP1	TACTACTACCTGATAGGAAGCAA
	4	JMTV-s4-5GSP1	CCTAGTCCATTGGTTGATTCTTA
Takachi virus	1	IMOI-Js1-F	CGTGAAGTATGGAAGTGAGGAAC
	1	IMOI-Js1-R	CCCTTTGAGATGCCTTTTAAGTT
	1	TAKV-s1-3GSP1	ACCTGCTGTTGACACCAGACAAA
	1	TAKV-s1-5GSP1	TCGAGGGCTTGTCACCCGAGTAA
	2	IMOI-Js2-F	CCTACGAAAAAGCAATAGAGCAA
	2	IMOI-Js2-R	GACGTCTAAAAGCGGGTAAATCT
	2	TAKV-s2-3GSP1	GAACGACTACTAGACTGCGGAAA
	2	TAKV-s2-5GSP1	TTTCGTAGGACGATTGCGGCAAT
	3	IMOI-Js3-F	GAAACCTCATAATGGTGGCAATA
	3	IMOI-Js3-R	GGCTCATACCGCCTATTCTCTAT
	3	TAKV-s3-3GSP1	ACGACCAGGAACGGACCCGGAAA
	3	TAKV-s3-5GSP1	TGGTCGGCAGTAGCTCCATGAAA
	4	IMOI-Js4-F	TACTCTTCAGCCTGGCAGTTTAC
	4	IMOI-Js4-R	ACTGTCGTCTTCCATAGGTGGTA
	4	TAKV-s4-3GSP1	GGCAGCCGCATACGGAAGCGGAA
	4	TAKV-s4-5GSP1	ATGAGCAGGGTGAGGCCAGGTAT

Supplementary Table S3. Number of individuals of ticks used for RNA virome analyses in this study.

Tick species	Stage*	Collection site (month / year)								Total no. of individuals per tick species and stage
		Ozu, Ehime (9, 11, 12 / 2018)	Imabari, Ehime (6,7, 9-12 / 2019)	Imabari, Ehime (1-6 / 2020)	Wajima, Ishikawa (9, 10 / 2018)	Wajima, Ishikawa (4-11 / 2019)	Wajima, Ishikawa (6-8 / 2020)	Kaga, Ishikawa (9-11 / 2018)	Kaga, Ishikawa (4-7, 9-11 / 2019)	
Amblyomma testudinarium	AF	1	0	0	0	0	0	0	0	1
	AM	0	1	0	0	0	0	0	0	1
	N	37	15	20	0	0	0	0	1	73
	L	0	0	17	0	0	0	0	0	17
Haemaphysalis flava	AF	20	7	10	0	0	0	0	0	37
	AM	48	17	8	0	0	0	0	0	73
	N	585	605	336	65	81	47	186	232	2137
	L	0	0	7	0	0	0	0	0	7
Hae. formosensis	AF	3	18	0	0	0	0	0	0	21
	AM	9	17	8	0	0	0	0	0	34
	N	167	822	688	0	0	0	0	0	1677
	L	0	0	0	0	0	0	0	0	0
Hae. hystricis	AF	0	26	8	0	0	0	0	0	34
	AM	0	13	8	0	0	0	0	0	21
	N	12	56	318	0	0	0	2	0	388
	L	0	0	0	0	0	0	0	0	0
Hae. longicornis	AF	0	0	0	0	0	0	0	0	0
	AM	0	0	0	0	0	0	0	0	0
	N	16	2	2	4	26	53	12	25	140
	L	0	0	0	0	0	0	0	0	0
Hae. megaspinosa	AF	0	0	0	0	0	0	0	0	0
	AM	0	0	0	0	0	0	0	0	0
	N	0	0	0	0	0	0	0	4	4
	L	0	0	0	0	0	0	0	0	0
Haemaphysalis sp.	AF	0	0	0	0	0	0	0	0	0
	AM	0	0	0	0	0	0	0	0	0
	N	0	0	0	0	0	0	0	0	0
	L	170	112	10	0	0	0	0	0	292
Ixodes ovatus	AF	0	0	0	0	0	0	0	0	0

	AM	0	0	0	0	0	0	0	0	0
	N	0	0	0	0	0	0	0	2	2
	L	0	0	0	0	0	0	0	0	0
I. persulcatus	AF	0	0	0	0	0	0	0	0	0
	AM	0	0	0	0	0	0	0	0	0
	N	0	0	0	0	0	1	0	0	1
	L	0	0	0	0	0	0	0	0	0
I. turdus	AF	0	0	2	0	0	0	0	0	2
	AM	0	0	0	0	0	0	0	0	0
	N	0	1	33	0	0	0	0	1	35
	L	0	11	0	0	0	0	0	0	11
Total no. of individuals per collection site		1068	1723	1475	69	107	101	200	265	

*Adult female (AF), Adult male (AM), Nymph (N), and Larva (L).
